# Histopathological responses and recovery in gills and liver of Nile tilapia (*Oreochromis niloticus*) exposed to diesel oil

**DOI:** 10.1016/j.toxrep.2022.10.005

**Published:** 2022-10-08

**Authors:** Jabed Hasan, Syed Rubaiyat Ferdous, Shams Binte Abi Rabiya, Md Firoj Hossain, AKM Munzurul Hasan, Md Shahjahan

**Affiliations:** a Laboratory of Fish Ecophysiology, Department of Fisheries Management, Bangladesh Agricultural University, Mymensingh 2202, Bangladesh; b Department of Fisheries Biology and Genetics, Bangladesh Agricultural University, Mymensingh 2202, Bangladesh

**Keywords:** Ecotoxicology, Oil pollution, Petroleum hydrocarbons, Tilapia

## Abstract

Water pollution due to crude oil has become one of the major means of pollution lately. We experimented to study to what extent different concentrations of diesel oil can distress the gills and liver of the affected fish. Two groups of Nile tilapia (*Oreochromis niloticus*) were exposed to 0.1 ml/l and 0.5 ml/l diesel oil for seven days and then kept in pollutant-free water for 14 days to scrutinize how much they can recover. A control group has also maintained during the experiment. Several histo-pathological abnormalities were observed in gills including deformed pillar system, clubbed tips in the secondary lamellae, hyperplasia of the epithelial cells, aggregation of cells of the primary lamellae, fusion of secondary lamellae, telangiectasia and lamellar aneurysms. Though almost similar level of aberration was observed in the lower and higher treatment group, fish treated with lower concentrations were quick to recover. When it comes to the liver, fish treated with 0.1 ml/l diesel showed mild necrosis, patchy degeneration, hypertrophy nucleus and which eventually recovered after 14 days of the recovery period, whereas fish treated with 0.5 ml/l diesel showed moderate to severe abnormalities in almost all cases and the recovery was less but pattern was observed. The experimental study concluded that the higher the exposure to diesel oil, higher incidences of major health problems are recorded, seriously piercing the healing system of Nile tilapia.

## Highlights

• The Nile tilapia was exposed to 0, 0.1 and 0.5 ml/l diesel oil for 7 days and then diesel oil free water for 14 days.
• Several histopathological abnormalities were observed in gills and liver of the exposed fish.
• Fish treated with lower concentrations were quick to recover.
• High exposure of diesel oil cause a major health problem and pierce the healing system.

## Graphical Abstract

**Figure:**
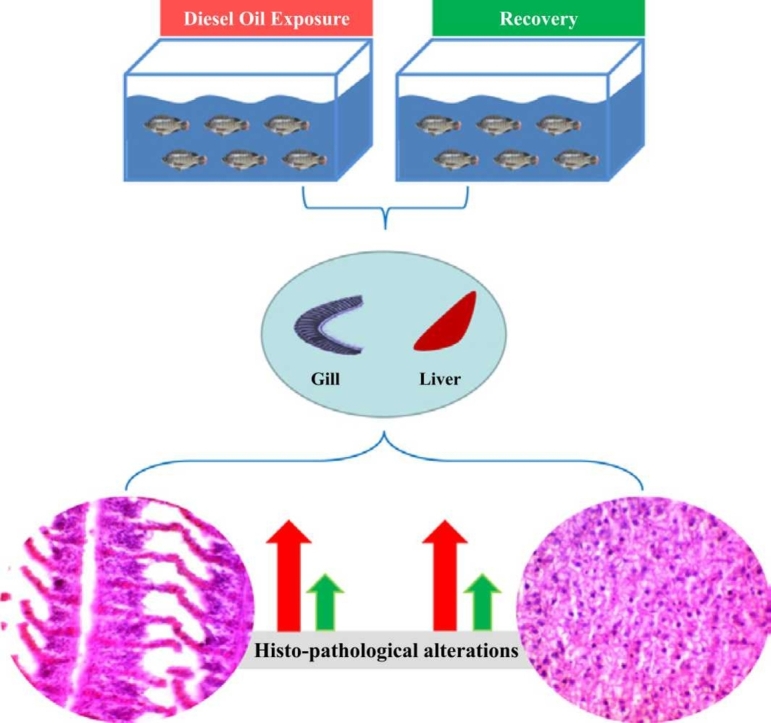

## 1 Introduction

Aquatic pollution due to anthropogenic contaminants has become a major concern around the globe. Diesel is a refined hydrocarbon and widely used as engine fuel. Because of urbanization and industrialization and an increased number of vehicles, petroleum-related activities have become commonplace. Diesel fuel and its elements are among the most commonly encountered toxins by people and wildlife existing in urban and rural areas. It is accountable for sub-lethal and long-lasting effects on fish [1]. Coming in contact with crude oil and products can prompt a variety of lethal warning signs in experimental animals. Petroleum hydrocarbons can act as a facilitator and induce free radical generation in fish [2]. They can easily be mixed with water and tend to stay in the water body for a long period. The chemicals in diesel can be stored in different organs of an animal and hard to eliminate. Petroleum hydrocarbons can be up-taken by aquatic organisms and accumulate in tissues 10–100 times greater than in water [3]. There are some reports which discussed the deleterious effect of diesel oil on the brain, physiology, gill anatomy, and reproduction of fish [4], [5], [6].

Fish gill is multifunctional and responsible for important work like respiration, acid-base balance, osmoregulation, and nitrogenous excretion [7]. Though fish gill's anatomy differs from one species to another, the cells involved in gill structure are very similar [8]. Gills are the main route of absorbing waterborne pollutants like hydrocarbons by aquatic organisms [9], [10], [11], [12], [13], [14]. Hence, studying their morphology can be served as a bio-indicator in environmental assessment [15].

The liver is one of the most vital organs in a fish body that is responsible for metabolism and accumulating, excreting, and transforming toxic elements [11], [14], [16], [17]. The liver is vulnerable to toxin-induced damage and reacts differently depending on the type and severity of the toxicants [18]. Because of its role in detoxification liver plays a significant part in eliminating harmful chemicals from the bloodstream [19]. Therefore, studying liver histology can be useful in evaluating the harm caused by toxicants. Moreover, histopathological examinations have been proved to be a profound tool to identify the effects of chemical compounds within the target organ of aquatic animals in laboratory tests [20], [21], [22].

The Nile tilapia (*Oreochromis niloticus*) is one of the most widely cultured freshwater fish across the world [23], [24]. It is popular because of its fast growth rate, easy management, omnivorous nature, hardiness, resistance to low dissolved oxygen, and enjoyable flavor [25]. Nile tilapia is a common model organism in the field of ecotoxicology and is suitable for monitoring toxic-induced changes in fish health [26], [27], [28]. Several studies addressed the effect of crude oil on the different organs for example gill, liver, and kidneys through histology. However, the study is still inadequate and to our knowledge, no research has been done to study the recovery of the affected organ if the pollutants are removed. The present study focused on diesel-induced histopathology of the vital organs like the gills and liver. In addition, the toxic effect on alteration in histopathology due to diesel oil along with the recovery pattern after the withdrawal of diesel was observed.

## 2 Materials and methods

### 2.1 Experimental fish

Healthy Nile tilapia fingerlings with a good physical appearance were collected from BFRI (Bangladesh Fisheries Research Institute), Mymensingh. The average body weight of the tilapia was 6.0 g and the mean length was 8.0 cm. The collected fish were first kept in the laboratory for 15 days in natural photo-regime conditions in fresh glass aquaria with 30 L of water for adjusting the fish to the laboratory environment. They were served with commercial fish feed (Mega fish feed, Bangladesh) two times a day around 9.00 AM and 5.00 PM by 5% of the entire body weight during the adaptation period. The experimental method and fish used in the research have been approved by the Animal Welfare and Ethical Committee, Bangladesh Agricultural University, Mymensingh.

### 2.2 Exposure assessment

The fingerlings of Nile tilapia were treated with three different doses (0.0, 0.1, and 0.5 ml/L) of diesel oil for 7 days. The selected concentrations were based on research on tilapia by [26]. Water was replaced every 24 h and anticipated concentrations of diesel oil were adjusted accordingly. The fish were provided with food 2 times a day. After 7 days of treatment, eight fish (n = 8) were sacrificed to collect gills and liver from each aquarium. Soon after the collection, the gills and liver were fixed in Bouin’s fluid for 24 h. Then the gills and liver were preserved in 70% alcohol and were kept in a refrigerator at 4 °C for histological analysis.

### 2.3 Recovery assessment

Once the exposure period was over the remaining fish were kept in completely diesel-free water for 14 days to scrutinize the recovery pattern of their gill and liver. The water was exchanged daily and the fish were provided with food 2 times a day. After 14 days, eight fish (n = 8) were sacrificed and their gills and liver were preserved same as exposure to study the recovery assessment.

### 2.4 Histo-pathological studies

The preserved gills and livers were dehydrated in ethanol and cleared using pure chloroform. After that, the tissues were embedded in paraffin wax with a melting point of 60–70 °C, and blocks were prepared for sectioning. The blocks were cut maintaining about 5 µm width and were fixed on slides. The samples were then left for air drying the whole night. Finally, the samples were stained with Eosin and Hematoxylin and mounted in DPX for extending their durability. The stained slides were studied under a digital light microscope (Micros, MCX100, Austria) at 40 × magnifications, and photographs were taken by a digital microscopic camera (AmScope MA1000). Atypical tissues and histological abnormalities were detected carefully in the experiment's control, treated, and recovery group.

### 2.5 Data analysis

The semi-quantitative histopathological alterations were examined as labeled by Mishra and Mohanty (2008). The mean prevalence of each histopathological parameter was categorized as no abnormalities (–, 0% of sections), mild abnormalities (+, <10% of sections), moderate abnormalities (++, 10–50% of sections) and severe abnormalities (+++, >50% of sections).

## 3 Results

### 3.1 Histo-pathological alterations of gills after exposure to diesel oil

**Fig. 1:**
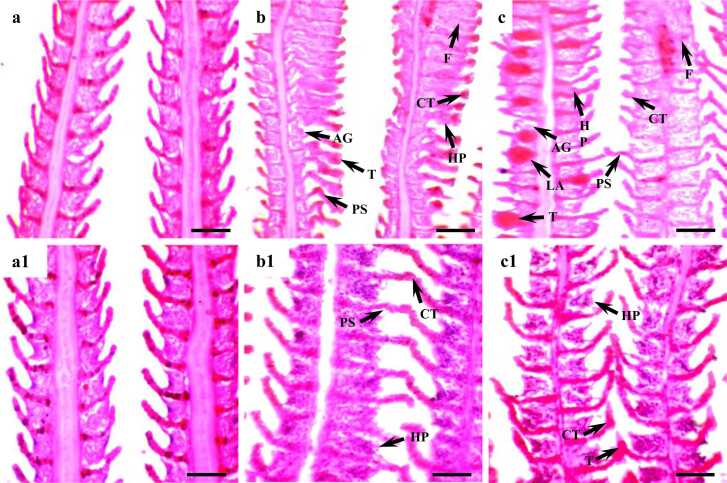
Histological section of gills of Nile tilapia (*Oreochromis niloticus*) exposed to different concentrations of diesel oil at 7 days (a = 0; b = 0.1 and c = 0.5 ml/L) and recovery at 14 days (a1, b1 and c1). PS; deformed pillar system, CT; clubbed tips, HP; hyperplasia, F; fusion, AG; aggregation of cells, T; telangiectasis and LA; lamellar aneurysms. H & E stain. Scale bar: 50 µm. Magnification 40 × .

Fish in the control group did not show any aberration throughout the experiment. Gill filaments and gill lamellae were well synchronized with inter-lamellar space. Fish exposed to both 0.1 ml/l and 0.5 ml/l diesel oil showed severe (>50%) deformed pillar system, clubbed tips in the secondary lamellae, and fusion of secondary lamellae (Fig. 1 & Table 1). Hyperplasia of the epithelial cells was moderate (10–50%) in both treatment groups. Telangiectasia was also found in both groups but was severe in the 0.5 ml/l group. Aggregation of cells of the primary lamellae was mild (<10%) in both of the treatment groups. Fish exposed to 0.1 ml/l did not show any lamellar aneurysms whereas it was severe in the 0.5 ml/l treatment group.

**Table 1:** Changes in gills morphology of Nile tilapia exposed to different concentrations of diesel oil for 7 days and recovery for 14 days.

Parameters	Diesel oil (ml/L)	Exposure (7 days)	Recovery (14 days)
Deformed pillar system	0.0	−	−
	0.1	+ ++	+ +
	0.5	+ ++	+ +
Clubbed tips in the secondary lamellae	0.0	−	−
	0.1	+ ++	+
	0.5	+ ++	+ +
Hyperplasia of the epithelial cells	0.0	−	−
	0.1	+ +	+
	0.5	+ +	+ +
Fusion of secondary lamellae	0.0	−	−
	0.1	+ ++	+ +
	0.5	+ ++	+ ++
Aggregation of cells of the primary lamellae	0.0	−	−
	0.1	+	+
	0.5	+	+
Telangiectasis	0.0	−	−
	0.1	+ +	+
	0.5	+ ++	+ +
Lamellar aneurysms	0.0	−	−
	0.1	−	−
	0.5	+ ++	+

− , None (0%); + , mild (<10%); + +, moderate (10–50%); + ++ , severe (>50%)

### 3.2 Recovery of histo-pathological alterations of gills

Recovery of histo-pathological alterations of gills was measured after treating them for 14 days with diesel-free freshwater. Fish exposed to the 0.1 ml/l treatment group displayed recovery from all the abnormalities they showed after diesel exposure. The only exception was the aggregation of cells of the primary lamellae which did not exhibit any recovery. However, none of the gill abnormalities completely disappeared from the 0.1 ml/l treatment group. Almost similarly the abnormalities for example deformed pillar system, clubbed tips in the secondary lamellae, hyperplasia of the epithelial cells, telangiectasis in the gills of fish exposed to 0.5 ml/l diesel oil healed in the recovery period. The only two exceptions were a fusion of secondary lamellae and hyperplasia of the epithelial cells which did not show any recovery during the recovery period. Recovery of lamellar aneurysms was most evident in this group. However, none of the samples showed complete recovery. One simple pattern was observed in most cases of recovery in both of the treatment groups which is that the abnormalities of gill structure became moderate where damages were severe and the abnormalities became mild where the damages were moderate (Fig. 1 & Table 1).

### 3.3 Histo-pathological alterations of liver after exposure to diesel oil

**Fig. 2:**
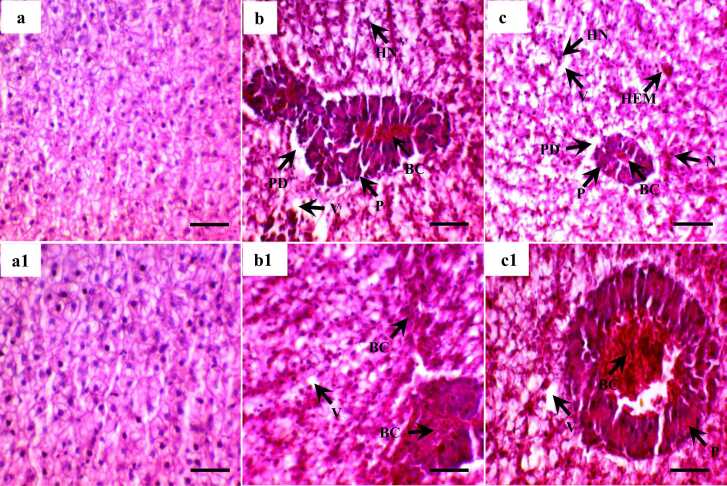
Histological section of liver of Nile tilapia (*Oreochromis niloticus*) exposed to different concentrations (a = 0; b = 0.1 and c = 0.5 ml/L) and recovery at 14 days (a1, b1 and c1). BC; blood congestion, V; vacuolization, HEM; hemorrhage, PD; patchy degeneration, N; necrosis, HN; hypertrophy nucleus and P; pancreatic tissue. H & E stain. Scale bar: 50 µm. Magnification 40 × .

No incongruities in livers were noticed in the control group throughout the experiment. Liver anomalies were greater with the increase in the diesel concentration. Several histopathological deformities were detected, including vacuolization, necrosis, patchy degeneration, blood congestion, hemorrhage, hypertrophy nucleus (Fig. 2 & Table 2). No severe abnormalities were found in the 0.1 ml/l treatment group. This group showed moderate vacuolization, blood congestion, hemorrhage, mild necrosis, patchy degeneration, and hypertrophy nucleus. Whereas the 0.5 ml/l treatment group showed severe vacuolization, necrosis, hemorrhage and moderate patchy degeneration, blood congestion, and hypertrophy nucleus.

**Table 2:** Changes in histo-morphology of liver of Nile tilapia exposed to different concentrations of diesel oil for 7 days and recovery for 14 days.

Parameters	Diesel oil (ml/L)	Exposure (7 days)	Recovery (14 days)
Vacuolization	0.0	−	−
	0.1	+ +	+
	0.5	+ ++	+ +
Necrosis	0.0	−	−
	0.1	+	−
	0.5	+ ++	+ +
Patchy degeneration	0.0	−	−
	0.1	+	−
	0.5	+ +	+
Blood congestion	0.0	−	−
	0.1	+ +	+
	0.5	+ +	+
Hemorrhage	0.0	−	−
	0.1	+ +	+
	0.5	+ ++	+ +
Hypertrophy nucleus	0.0	−	−
	0.1	+	−
	0.5	+ +	+

− , None (0%); + , mild (<10%); + +, moderate (10–50%); + ++ , severe (>50%)

### 3.4 Recovery of histo-pathological alterations of liver

Recoveries were evident in all the samples of 0.1 ml/l treatment after 14 days of the recovery period. Samples that showed mild necrosis, patchy degeneration, and hypertrophy nucleus recovered completely. However, none of the samples with moderate abnormalities recovered completely but showed an obvious recovery pattern. All the samples from the 0.5 ml/l treatment group also demonstrate recovery after the 14 days recovery period. However, none of the samples were fully recovered in this period but severe abnormalities became moderate and moderate abnormalities lessened to mild (Fig. 2 & Table 2).

## 4 Discussion

The presence of excess diesel oil in water can seriously damage the vital organs of aquatic creatures and hamper growth, reproduction rate, and survival rate [29]. Toxicants in water can easily come in direct contact with the gill surface and cause damage. Diesel contains polycyclic aromatic hydrocarbons which affect the respiratory system of fish for an extended period [30]. The degenerative changes were comparatively more pronounced in gill than in liver tissues. This may be because gills remain in direct contact with the pollutants of water [31]. Gills are the primary path to absorbing waterborne contaminants (including hydrocarbons) by aquatic organisms [9]. Crude oil hydrocarbons have been shown to stimulate structural abnormalities in the branchial respiratory epithelium [32]. In this experiment, we found several histo-pathological abnormalities in gill including a deformed pillar system, clubbed tips in the secondary lamellae, hyperplasia of the epithelial cells, fusion of secondary lamellae, aggregation of cells of the primary lamellae, telangiectasis, and lamellar aneurysms. It has been reported that oils containing hydrocarbons caused several damages to the gills including hemorrhage, rupture of the pillar cell, hypertrophy, hyperplasia of epithelial cell, and fusion of secondary gill lamellae of juvenile rabbit fish *Siganus canaliculatus* [33]. Several histo-pathological abnormalities in *Clarias gariepinus* due to the exposure to diesel oil [29], supported the present findings. It has been also reported histopathological gill damage due to the exposure of crude oil in yellow perch *Perca flavescens*, goldfish (*Carassius auratus*) and *Prochilodus lineatus* [34], [35]*.* In another study gill histopathological alterations including fusion of secondary gill lamellae, aneurism, breakdown and abnormality of the secondary lamella, proliferation, and hypertrophy of Milkfish (*Chanos chanos*) were found in acute toxicity of diesel oil which supports our study [36]. In this experiment, the damage level of the 0.1 ml/l treatment group was almost equal to the damage level of the 0.5 ml/l treatment group. However, in the case of lamellar aneurysms, the 0.1 ml/l treatment group showed zero abnormalities but in the case of the 0.5 ml/l treatment group, it was severe. Though it indicates that a higher concentration of diesel pollutants may cause serious damage compared to a lower concentration, we cannot say that lower dose pollutants are less harmful to gill as the other abnormalities showed an almost similar pattern regardless of the concentration of diesel. Aneurysms might have resulted from the breakdown of pillar cells and the collapse of vascular integrity [37]. This indicates that fish exposed to a lower dose of diesel pollutants may have the ability to keep their pillar cell and vascular integrity intact but if the concentration of pollutants is higher the defense system may break down. Though we found an almost similar level of aberration in the lower and higher treatment group, fish treated with lower concentrations were quick to recover. It is possible that the overall health condition of the group treated with a higher concentration of diesel was more severe compared to the group treated with a lower dose which helped the 0.1 ml/l treatment group to heal quickly. As the gills have direct contact with the water it remains extremely vulnerable to the pollutants present in water which might be a possible reason for the same amount of damage to both higher and lower doses of diesel.

The liver plays important role in detoxification and biochemical transformation and aberration in the liver cells may result from the pollutants present in water [16], [38], [39]. The liver is also one of the most susceptible organs affected by the water-soluble fraction of crude oil [40]. Because of its high sensitivity to pollutants, the liver can be used as an indicator for environmental monitoring [41]. Thus, changes in its structure can be used in the assessment of the well-being of fish [42]. In the current study, histo-pathologies like vacuolization, necrosis, patchy degeneration, blood congestion, hemorrhage, hypertrophy nucleus were found in the fish exposed to 0.1 ml/l and 0.5 ml/l diesel for 7 days. More or less similar histopathological abnormalities like deterioration of endothelial lining cells, inflammation of hepatocytes and diffuse necrosis, and hemolysis within the blood vessels were observed in the liver of *Channa punctatus* exposed to diesel fuel [43]. In another study, nuclear hypertrophy, cellular hypertrophy, vacuolation, and blood congestion were reported in the liver of *Prochilodus lineatus* exposed to diesel oil [35], which is consistent with our study. Histopathological abnormalities in the liver were also detected in Flounder (*Pleuronectes americanus*) found close to an oil refinery which exhibited several histological lesions caused because of the effect of the oil on these fish [44]. Unlike gill histopathology, the abnormalities in the liver were great in the 0.5 lm/l treatment group compared to the 0.1 ml/l treatment group. This proves that a higher concentration of pollutants can cause serious damage to the liver. Fish treated with 0.1 ml/l diesel showed mild necrosis, patchy degeneration, hypertrophy nucleus and which eventually recovered after 14 days of the recovery period. This finding indicates the concentration of the pollutants is light the fish may recover from some of the abnormalities. None of the abnormalities in 0.1 ml/l were severe. Whereas fish treated with 0.5 ml/l diesel showed moderate to severe abnormalities in almost all cases. Necrosis observed in the liver probably resulted from the excessive work required by the fish to eliminate the crude oil from its body through detoxification. The failure of fishes to produce new liver cells may also have given rise to necrosis. Hemorrhage is an outcome of blood channel disruption and is a symptom of severe physical impairment. Vacuolization, patchy degeneration, blood congestion, and hypertrophy nucleus can be attributed to the direct toxic effect of diesel oil since the liver is the place of detoxification of all sorts of pollutants and chemicals. However, recovery was quite obvious in this treatment group as well. Unlike the 0.1 ml/l treatment, none of the abnormalities disappear completely as the damage level was more intense in this group, indicating the severity of the more concentrated pollution. None of the severe aberrations degraded to mild ones either. This indicates the possibility of long-term damage due to diesel pollution. As we observed a recovery pattern after 14 days of the recovery period, it is possible that the 0.5 ml/l treatment group may recover in the long run. However, a more rigorous study is needed to prove that the damages are not permanent and they will recover given that the water becomes completely pollutant-free.

## 5 Conclusion

The present experiment tells that diesel oil have a severe effect on the gills and liver of Nile tilapia. It also reveals that fish gills are more susceptible to toxins compared to an internal organ as it remains in direct contact with contaminated water. It also revealed that recovery of the gill is rapid regardless of the severity of the damage if the concentration of the pollutants is low. It showed that fish liver can withstand a low level of diesel toxicants and can recover mild abnormalities quickly but is severely vulnerable to a high concentration of pollutants. We suggest more research regarding this matter at the molecular level to get a more precise result in case of damage and recovery.

## Ethics approval

The total procedure conducted in this experiment was approved by the animal care and use committee of Bangladesh Agricultural University, Mymensingh (Approval Number: BAU-FoF/2020/004).

## CRediT authorship contribution statement

Jabed Hasan and Syed Rubaiyat Ferdous performed the experiment, collected data and drafted the manuscript. Shams Binte Abi Rabiya, Md Firoz Hossain and AKM Munzurul Hasan participated in data collection. Md Shahjahan assisted in the experimental design and edited the manuscript. All authors reviewed and approved the final manuscript.

## Declaration of Competing Interest

The authors declare that they have no known competing financial interests or personal relationships that could have appeared to influence the work reported in this paper.

## Data Availability

The data that support the outcomes of this study are available on request from the corresponding author [M Shahjahan].
